# Journey Towards Piloting *Helicobacter pylori* Screen‐and‐Treat to Address Health Inequities in Aotearoa New Zealand

**DOI:** 10.1111/hel.70123

**Published:** 2026-04-03

**Authors:** Andrea Teng, James Stanley, Melissa McLeod

**Affiliations:** ^1^ University of Otago Wellington New Zealand

## Abstract

**Background:**

Māori and Pacific peoples in Aotearoa New Zealand (Aotearoa) face the greatest barriers to healthcare access, poorest health outcomes, and disproportionate levels of deprivation.

**Methods:**

This paper outlines the (1) epidemiology of *H. pylori* and its sequelae, (2) inequities in the current *H. pylori* approach, (3) research into screen‐and‐treat, and (4) our recommendations going forward.

**Results:**

There are stark ethnic differences in prevalence of *H. pylori* infection and its sequelae in Aotearoa—with higher *H. pylori* infection prevalence and gastric cancer incidence and higher numbers of hospitalisations for peptic ulcer in Māori, Pacific, and Asian ethnicities than in European. The opportunistic approach taken to *H. pylori* testing has created inequities. Māori and Pacific are less likely to be tested for *H. pylori* than European, despite the higher prevalence of infection in these populations. In Aotearoa, a targeted screen‐and‐treat approach has been shown to be more cost‐effective than a population–wide strategy.

**Conclusion:**

There is an urgent need to introduce a screen‐and‐treat pilot in Aotearoa, which should be led by Māori. Piloting of screen‐and‐treat is useful for evaluating invitation, testing and treatment strategies. Further *cost* –effectiveness modeling could support the evaluation of more specific targeting, test choice, and treatment choice where input data allow.

## 1 Introduction

Aotearoa New Zealand (Aotearoa) is a South Pacific nation with 5 million people. Māori are the Indigenous population (17.8% in 2023 census), with European colonizers settling since the early 19th century (currently 67.8% of the population) and Asian (17.3%) and Pacific peoples (8.9%) arriving in more recent waves of migration. The healthcare system is predominantly publicly funded through general taxation, centrally managed and universal, but with co‐payment generally required for primary care visits. Māori and Pacific peoples are disproportionately represented in the most deprived areas, facing the greatest barriers to healthcare access and the poorest health outcomes. Ethnic inequities exist across almost all diseases, and throughout the healthcare pathway for the Indigenous Māori population. This paper will outline in Aotearoa the (1) epidemiology of *H. pylori* and its sequalae, (2) inequities in the current *H. pylori* management approach, (3) research into screen‐and‐treat, and (4) recommended future research directions.

## 2 Epidemiology of *H. pylori* and Its Sequalae

The epidemiology of *H. pylori* in Aotearoa is characterized by stark ethnic differences in prevalence: in the asymptomatic population, the rate of seropositivity in Pacific peoples has been reported as three times that in European people, and the rate of seropositivity in Māori people is twice that in European people (based on available studies from before 2000) [1]. Positivity rates from routine *H. pylori* testing also consistently follow this pattern; for example, in one predominantly primary care‐based study (2015–2018, Northern health region) stool antigen test (SAT) positivity rates were 45% in Middle Eastern, Latin American, or African, 39% in Pacific, 29% in Asian (which includes both East Asian and South Asian ethnicities), 24% in Māori and 17% in European populations [2], with Māori and Pacific experiencing the lowest rates of testing. Table 1 summarizes the consistent pattern of ethnic inequities, and also suggests that within ethnicity groups, active infection (via SAT) is more commonly identified in symptomatic patients tested in the Northern health region than in Canterbury, South Island.

**TABLE 1:** Summary of ethnic inequities in *H. pylori* infection and its sequalae in Aotearoa New Zealand.

	Māori	Pacific	Asian	European
Population size in 2023^a^	887,493	442,632	861,576	3,383,742
*H. pylori* testing rates (per 1000 person‐years, Auckland/Northland) [2]	6.4	7.2	20.8	11.8
Asymptomatic: relative rates of *H. pylori* seropositivity (pre‐2000) [1]	1.9	3.4	—	1.0 (ref)
Symptomatic *H. pylori* positivity rate (serology), %				
Auckland/Northland, 2015–2018 [2]	19	35	23	12
Canterbury, 2013–2018 [3]	21	38	28	8
Symptomatic *H. pylori* positivity rate (stool antigen test), %				
Auckland/Northland, 2015–2018 [2]	24	39	29	17
Canterbury, 2013–2018 [3]	13	29	23	5
Canterbury, 2022–2023 [4]	12	33	24	8
Peptic ulcer hospitalization rate (per 100,000 person‐years, 2015–2018)^b^	51	63	22	15
Gastric cancer incidence (per 100,000 person‐years, 2017–2021)^c^	11.1	13.9	5.5	4.1
Gastric cancer mortality rate (per 100,000 person‐years, 2017–2021)^c^	7.4	8.5	3.0	2.6

^a^ Individuals may identify to more than one ethnicity.

^b^ Standardized to the 2001 Māori census population.

^c^ Standardized to the World Health Organization population standard, Te Whatu Ora, available from https://tewhatuora.shinyapps.io/cancer‐web‐tool/, accessed on 30 June 2024.

### 2.1 Sequalae

The age‐standardized incidence rates of gastric cancer in Aotearoa are low in the international context, at 8 per 100,000 person‐years in males and 4 per 100,000 person‐years in females in 2015–2019, when age‐standardized to the World Health Organization (WHO) world standard population [5]. The equivalent crude rates are 11 per 100,000 in males and 6 per 100,000 in females, with an overall rate of 8.5 per 100,000 (2015–2019) [5].

**FIGURE 1:**
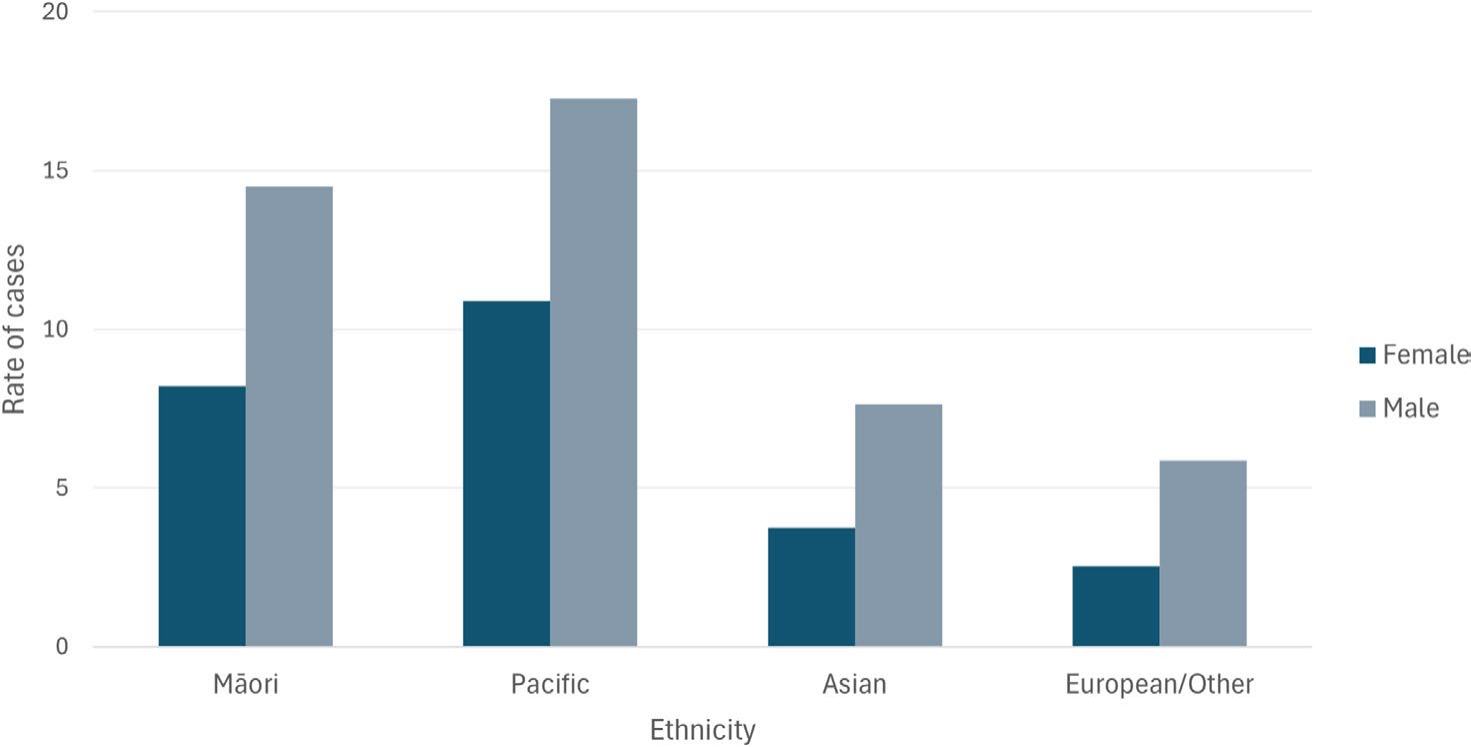
Rate of gastric cancer registrations in 2017–2021 by ethnicity. Rate is per 100,000 person‐years and is age‐standardized to the World Health Organization world standard population. Rates were downloaded from (https://tewhatuora.shinyapps.io/cancer‐web‐tool/, accessed on 30 June 2024).

However, there are stark ethnic differences in gastric cancer incidence and mortality, with Māori and Pacific experiencing incidence rates that are 3.4, 2.4 (males), 5.8, and 3.7 (females) times those of European/Other people respectively [6], with the rates of gastric cancer in Asian people being somewhere in between Māori/Pacific and European/Other [7]. Current gastric cancer incidence rates in Aotearoa (Figure 1, 2017–2021, age standardized to WHO standard population) are highest in Māori (15 and 8 per 100,000 person‐years in males and females respectively) and Pacific peoples (17 and 11), lower in Asian (8 and 4) and lowest in European/Other (6 and 3). Māori with gastric cancer are also more likely than non‐Māori to have non‐cardia gastric cancer [8] (about 80% vs. 50%, if overlapping and undefined types of gastric cancer are excluded) [9] and diffuse‐type gastric cancer [8].

**FIGURE 2:**
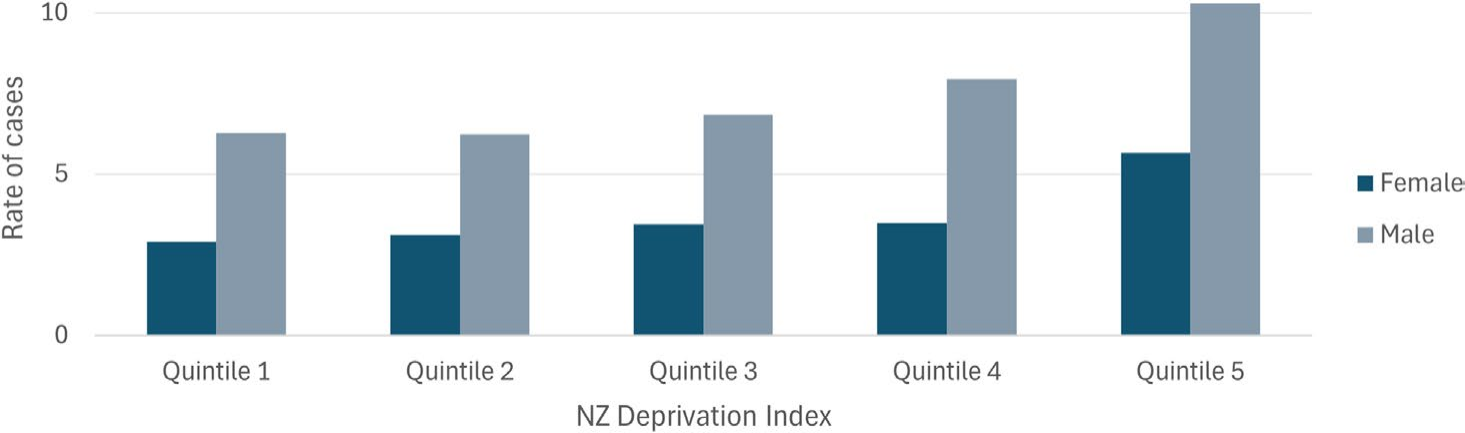
Rate of gastric cancer registrations in 2017–2021 by New Zealand Deprivation Index quintiles. Rate is per 100,000 person‐years and is age‐standardized to the World Health Organization world standard population. Rates were downloaded from (https://tewhatuora.shinyapps.io/cancer‐web‐tool/, accessed on 30 June 2024).

Gastric cancer incidence and mortality rates are also substantially higher in groups with the lowest socioeconomic position. For example, in 2006–2011, gastric cancer incidence rates were substantially higher in the lowest versus the highest equivalized household income quintile [10] (men: RR = 1.61, 95% confidence interval (CI): 1.03–2.56; women: RR = 1.81, CI: 1.00–3.29). Using area‐based deprivation (2017–2021 period), gastric cancer incidence rates were again substantially higher for those living in areas with the highest compared to the lowest quintile of deprivation, ie, 10 and 6 per 100,000 for males and females respectively living in areas with the highest levels of deprivation, compared with 6 and 3 per 100,000 for males and females in people living in areas with the lowest levels of deprivation (Figure 2).

The incidence of peptic ulcers also varies by ethnicity. Age‐standardized rates of hospital admission for peptic ulcer in Aotearoa [7] (2015–2018, age‐standardized to 2001 Māori census population) are 3.5 times higher in Māori (50.8 per 100,000 population; 95% CI: 47.5–54.4) and 4.3 times higher in Pacific (63.1 per 100,000 population; 95% CI, 57.9–68.6) compared with European (14.6 per 100,000 population; 95% CI, 13.8–15.4), with Asian having intermediate admission rates (21.8 per 100,000 population; 95% CI, 19.7–24.1).

Differential rates of *H. pylori* are the largest contributor to inequities in gastric cancer incidence in Aotearoa [11], and this is likely also the case for peptic ulcers. The screen‐and‐treat approach for *H. pylori* is expected to be a useful tool to address existing gastric cancer inequities by ethnicity, and possibly for groups with low socioeconomic position as well.

## 3 Gastric Cancer Risk Factors

In addition to chronic *H. pylori* infection, other risk factors for gastric cancer in Aotearoa are strongly patterned by ethnicity and socioeconomic position, as shown by the population prevalence of smoking, obesity and hazardous drinking reported by the New Zealand Health Survey [12]. In 2022/2023, 17% of Māori, 6% of both Pacific and European/Other, and 3% of Asian adults smoked every day. Obesity rates were 67% in Pacific, 48% in Māori, 32% in European/Other, and 14% in Asian adults. Hazardous drinking rates were 25% in Māori, 22% in Pacific, 17% in European/Other, and 5% in Asian adults. These risk factors were also more common in adults living in the highest deprivation areas (unadjusted for ethnicity).

## 4 Hereditary Risk

Higher rates of gastric cancer incidence in Māori, particularly among younger Māori, have also been thought to be caused by an increased propensity towards mutations of the *CDH1* gene in particular family groups. A study has estimated that 6% of Māori with advanced gastric cancers have a *CDH1* mutation [13], with higher rates found in younger age groups and in people diagnosed with diffuse‐type cancers. The rate of this mutation among European in Aotearoa with gastric cancer is less clear.

The impact of inherited genetic mutations appears to be particularly compounded by the presence of chronic *H. pylori* infection. Recent evidence from Japan shows an interaction between nine germline pathological variants and *H. pylori* infection. The lifetime risk of gastric cancer was strongly elevated when both *H. pylori* infection and one of these variants were present [14], resulting in a lifetime risk of gastric cancer of 45%, compared to < 5% in carriers of one of these variants with no *H. pylori* infection, and a lifetime risk of 14.4% in people with *H. pylori* infection who were not carriers of any of these variants.

## 5 Inequities in the Current *H. pylori* Approach

The opportunistic approach to *H. pylori* testing in Aotearoa creates inequities, which are then amplified through unequal and inequitable treatment of positive *H. pylori* tests—despite the higher *H. pylori* prevalence and gastric cancer incidence.

### 5.1 Testing

In current primary care practice in Aotearoa, the funded test for active *H. pylori* infection is the stool antigen test (SAT). The SAT is recommended in patients who present with one of the following risk factors: [15] history of peptic ulcer, family history of gastric cancer, or dyspepsia, and one of the following factors: aged > 60 years, Māori, Pacific, Asian, or African ethnicity, or someone who originates from an area of high *H. pylori* prevalence (> 30%), for example, south Auckland, Porirua, the East Cape, or a low‐ and middle‐income country, including countries in Asia. Urea breath tests are not publicly funded and are rarely used [3]. Serology is still widely used in many regions to test or screen for *H. pylori*, for example being more commonly used than the SAT until the end of 2018 [3].

Despite the higher *H. pylori* prevalence in Māori, Pacific, Asian, and Middle Eastern or Latin American or African people and the availability of ethnicity‐specific guidelines, rates of testing remain disproportionately low for Māori and Pacific people [2, 3, 7] compared with European (who have low *H. pylori* infection prevalence) [7]. In general, Māori and Pacific experience several barriers to primary care access and have the highest levels of unmet health‐care needs [16]. Access to primary care in Aotearoa usually requires co‐payments (that differ between health‐care providers), and funding for primary care has been found to have embedded historical inequity and has ignored unmet needs, and services for Māori have been systematically underfunded [17]. These underserved ethnic groups have the highest rates of gastric cancer incidence and are expected to gain the most (per‐person) benefit from an *H. pylori* screen‐and‐treat approach, particularly if the approach is introduced with an equity focus [9].

### 5.2 Gastroscopy

Guidelines in Aotearoa recommend referring for gastroscopy [15], for example, when *H. pylori* treatment has failed, in people with persistent symptoms despite treatment, or people with risk factors, such as: a first presentation of dyspepsia in people aged ≥ 50 years (or aged ≥ 40 years in at‐risk ethnic groups), family history of gastric cancer onset < 50 years, severe or persistent dyspepsia despite treatment, previous peptic ulcer disease, coughing spells, or nocturnal aspiration. Despite ethnicity‐specific guidelines, there are greater ethnic disparities in the rates of gastroscopy testing (e.g., in the use of rapid urease tests) than primary care testing for *H. pylori* , suggesting even greater barriers to access to secondary care for Māori and Pacific compared with European [2, 3]. These referral criteria do not guarantee access to gastroscopy, which varies geographically.

### 5.3 Treatment

Current national treatment guidelines recommend triple therapy for 7–14 days with a proton pump inhibitor, amoxicillin, and clarithromycin (OAC) as first‐line treatment, with metronidazole as a potential substitute for either antibiotic [18]. Quadruple therapy is recommended as a second‐line treatment in cases of eradication failure and comprises 2 weeks of a proton pump inhibitor, bismuth, tetracycline, and metronidazole [18]. This advice varies from the Maastricht Consensus report, which recommends first‐line treatment with 14 days of bismuth quadruple therapy in areas where clarithromycin resistance is > 15% (or unknown) and susceptibility testing is not available [19]. However, there is an urgent need to further investigate primary clarithromycin resistance rates, including by ethnicity [20].

Updated local information about clarithromycin and antibiotic resistance rates and *H. pylori* eradication rates would make a valuable contribution to the case for revising treatment guidelines in Aotearoa [21]. This is particularly important given the increase in clarithromycin resistance globally. There is likely to be increasing resistance to first‐line *H. pylori* treatment in Aotearoa. In 2012, a small study (*n* = 73) carried out in a relatively high deprivation area, in patients with positive gastroscopy specimens, reported 49% metronidazole resistance and 16% clarithromycin resistance [22], and 35% eradication failure of first‐line treatment (with omeprazole/amoxicillin/clarithromycin) in Māori, Pacific, and Asian people. A 2021 meta‐analysis investigated antibiotic resistance of *H. pylori* in Australia and Aotearoa [21] and reported a doubling of primary resistance to clarithromycin to 16% (95% CI: 11–22) post‐2000 compared with pre‐2000.

Increasing antibiotic resistance and poor eradication rates make it vital to increase retesting for eradication effectiveness, i.e., 4–6 weeks after treatment is completed [20] in line with international guidelines [19]. Retesting is not in the current treatment guidelines in Aotearoa and its use remains low [2]. Retesting enables the use of second‐line therapy to improve eradication rates, and also could improve the usefulness of laboratory data for monitoring eradication rates. Work is underway to make retesting for eradication success a routine recommendation across the regional clinical pathways.

## 6 Ethnicity Data Quality

Accurate ethnicity data are crucial for equitable health care, and targeted screen‐and‐treat participation, but Māori are undercounted in health data [23]. Improved protocols are needed for consistent, accurate ethnicity data collection.

## 7 Screen‐and‐Treat in Aotearoa

In response to stark inequities in *H. pylori* and its sequalae in Aotearoa, the New Zealand Cancer Action Plan 2019–2029 sets out a plan to develop a strategy to address *H. pylori* infection in priority populations [24]. Gastric cancer is one of the top ten contributors to the life expectancy gap for both Māori and Pacific populations in Aotearoa (compared with European people), and thus is a priority in the public health system. Several research projects have been set up to support future implementation of *H. pylori* screen‐and‐treat in Aotearoa. The following sections report cost‐utility modeling, research projects that are underway and potential future research directions.

## 8 Cost‐Utility Modeling for Population and Targeted Screen‐And‐Treat Approaches

Cost‐utility monitoring was applied to the Aotearoa setting, using *H. pylori* and gastric cancer epidemiology from 2011 [9]. An important contribution of this work is a comparison in cost–effectiveness of screen‐and‐treat approaches between the Indigenous Māori population (who have a moderate risk of gastric cancer) and the remaining population (who have low rates of gastric cancer on average; this remaining population also includes groups with a high risk of gastric cancer, such as Pacific people, who are likely to be a small proportion overall). The following model inputs were applied at different rates for Māori and non‐Māori people: (i) proportion of cancer that is non‐cardia; (ii) coverage of testing; (iii) eradication rate of triple therapy; and (iv) seroprevalence. Two *H. pylori* screen‐and‐treat scenarios were evaluated based on the diagnostic test used: one analysis used serology (primary analysis), and the other used SAT. The most relevant SAT results are reported here, given that serology is not recommended for diagnosing infection.

The SAT scenario cost NZ$ 369 million and resulted in 15,300 quality‐adjusted life years (QALYs) gained in men and women aged 25–69 years, with lifetime follow‐up. This resulted in an incremental cost–effectiveness ratio of NZ$ 29,000 per QALY. If Māori alone were targeted, the cost would be NZ$ 49 million and 4200 QALYs would be gained, which equates to a better value incremental cost–effectiveness ratio of NZ$ 13,700 per QALY gained.

The screen‐and‐treat programme in the population had four times the absolute health gain (i.e., clinical effectiveness, QALYs) than targeting Māori alone, but at more than seven times the cost. The QALYs gained by Māori were even greater in equity analyses in which life expectancy was set to the same level as non‐Māori [9]. The greater cost–effectiveness in Māori people is likely to be similar in other groups with high rates of *H. pylori* and gastric cancer in Aotearoa.

Although the modeled programme in the whole population was cost‐effective, it was more cost‐effective with a targeted approach for Māori [9], which supports the recommendation that high‐risk groups would be a useful priority for implementation of this programme.

Further cost–effectiveness modeling would be useful to assess the costs and benefits of targeting additional high‐risk groups and the impact of different testing and treatment modalities, such as the use of different types of tests, different treatment choices, and the inclusion of gastroscopy for participants who have clinical indicators of potential gastric cancer. This is possible as more precise model inputs become available; for example, better estimates of *H. pylori* prevalence and eradication rates.

## 9 *H. pylori* Research in Aotearoa

Several research streams were in the field in mid‐2025, with the aim of informing the future implementation of an *H. pylori* screen‐and‐treat pilot/programme in Aotearoa. Studies, for example, are investigating: (i) community estimates of *H. pylori* prevalence and a subsequent management pathway in the *H. pylori* in Aotearoa New Zealand (ENIGMA) Study; (ii) a family‐based index‐case method focused on an Indigenous people, which is recruiting participants for *H. pylori* screen‐and‐treat (Puku Ora Feasibility Study); (iii) *H. pylori* antibiotic resistance rates using the polymerase chain reaction (PCR) for genetic markers and culture; (iv) the feasibility and acceptability of test modality types in Māori and Pacific.

### 9.1 *H. pylori* in Aotearoa New Zealand (ENIGMA) Study

The *H. pylori* in Aotearoa New Zealand Study (HpANZ/ENIGMA) is investigating the ethnicity‐specific distribution of *H. pylori* infection in New Zealand (using biological specimens), the risk factors for *H. pylori* infection, overlap of infection with risk factors for gastric cancer, markers of antibiotic resistance, and implications of the approach for future screen‐and‐treat, including *H. pylori* case management. The study finished recruiting in mid‐2025 and plans to report results in 2026.

HpANZ uses a cross‐sectional survey design with a community sample of 12–69‐year‐olds, with secondary sampling from past respondents of the New Zealand Health Survey. The aim was to include 1188 participants with equal numbers of Māori, Pacific, and the remaining groups of European/Other people, and equal numbers of people across 10‐year age groups.

Participation involves responding to a survey by phone, blood test at a local laboratory, and the option of submitting a stool sample (which has the added benefit of investigating a diagnosis of *H. pylori* infection). Any person with a positive serology test is followed up with a SAT sent to a person's address (if the participant had not already opted‐in for this test). Case management is organised by a research nurse at Kōkiri marae with retesting to assess eradication at 6 weeks after completion of treatment.

### 9.2 Puku Ora Feasibility Study

The Puku Ora Feasibility Study takes a Kaupapa Māori, holistic, strength‐based approach to test the feasibility of an approach that addresses the health inequities between Māori and non‐Māori people in Aotearoa in gastric cancer and bowel cancer and recruits family members. A combined screening approach is used to screen and treat for *H. pylori* and screen for bowel cancer using a single stool sample, in a Māori‐specific context for people aged 45–60 years. Education and encouragement are given to promote participation in the National Bowel Screening Programme, if not already participating. Participants older than 60 years or adults younger than 45 years are tested for *H. pylori* only. Recruitment has been completed.

### 9.3 Antibiotic Resistance Studies

Several studies in Aotearoa have been investigating *H. pylori* antibiotic resistance in different clinical contexts, in addition to the *H. pylori* in Aotearoa New Zealand (ENIGMA) Study. Rates of *H. pylori* antibiotic resistance in positive gastroscopy isolates have been investigated in the Wellington region [25]. This study recruited symptomatic patients undergoing gastroscopy who have had a positive rapid urease test. The aim was to inform more precise treatment choice to improve eradication rates. DNA extraction from rapid urease tests enabled clarithromycin resistance gene testing. Resistance rates were similar in Māori and Pacific patients (15%, CI: 5% to 36%) and other ethnic groups (16%, CI: 9% to 26%), though the small sample size (*n* = 84) limited the precision with which we can understand or compare resistance rates by ethnicity. Overall eradication success was 88% (CI: 80% to 93%). Another study is investigating *H. pylori* antibiotic resistance also in a clinical setting in another region (Auckland).

### 9.4 Acceptability and Feasibility of *H. pylori* Screening Tests

Health New Zealand | Te Whatu Ora is currently leading two studies investigating the acceptability and feasibility of screen‐and‐treat test modalities. A survey and interviews will ask Māori and Pacific adults about their views about *H. pylori* for gastric cancer prevention, including the acceptability of urea breath test (UBT), SAT, and serology testing, as well as invitation, management, and family‐index case approaches. The feasibility of setting up UBT in the health system is also being investigated.

## 10 Recommendations

The next and perhaps the most important step in addressing the vast and unequal burden of *H. pylori* infection and its sequelae will be the implementation of an *H. pylori* screen‐and‐treat pilot programme. Given the inequities that exist across almost all diseases and throughout the healthcare pathway for the Indigenous Māori population in Aotearoa, we recommend Māori need to lead the design of an *H. pylori* screen‐and‐treat programme. This section describes some of the information needs and outstanding questions [20] that can be addressed in the implementation of a screen‐and‐treat pilot and ultimately support the broader programme rollout in Aotearoa. A screen‐and‐treat pilot should investigate the feasibility of proposed approaches, develop standardised treatment and case‐management protocols, and incorporate system‐level evaluation for validation of implementation pathways.

### 10.1 Targeting

1. Consensus is needed on which high‐risk groups to screen‐and‐treat, or whether wider implementation is needed to the whole population. Targeting decisions could be informed by analysis of *H. pylori* prevalence, and peptic ulcer and gastric cancer rates by age, sex, and ethnicity, including subgroups (e.g., East Asian and South Asian people), socioeconomic position, country of birth, family history, and other potentially relevant factors.
2. How could screen‐and‐treat processes be extended to household members of positive cases detected through the initial inclusion criteria, and how feasible and effective is this approach?

### 10.2 Testing

1. Consensus is needed on choice of diagnostic test (balancing ethnicity‐specific acceptability, capacity of the health system, and costs); for example, SAT, UBT, or serology then SAT/UBT, including considerations for where and how these tests will be done.
2. What are the rates of reinfection in Aotearoa, and is subsequent follow‐up testing needed after eradication is confirmed?
3. What are the most acceptable *H. pylori* screen‐and‐treat strategies for Māori and Pacific people, and which methods of engagement would support co‐design and improve awareness and participation?

### 10.3 Treatment

1. What are the current levels of *H. pylori* treatment antibiotic resistance for current (and alternative) first‐line and second‐line therapies? ie, to inform appropriate national treatment guidelines:
  - choice of first‐line therapy (considering resistance rates);
  - introducing retesting as standard practice
2. Develop a plan for assessing who is high risk and should be referred for gastroscopy for diagnosis of gastric cancer. Will blood markers of gastric cancer risk be used? What are the service impacts of increased endoscopy demand for the diagnosis of cancer?

### 10.4 Programme

1. What is the effectiveness, equity outcomes and cost–effectiveness of different targeting, testing, treatment, and combination screening approaches in Aotearoa?
2. Information on the pros and cons of delivering treatment and retesting via primary care or other more centralized/telehealth processes.
3. Development of an equitable process for invitation to screening, participation, and follow‐up for treatment including support for language and cultural needs of participants.
4. How could the screen‐and‐treat approach be integrated with other screening programmes: for example, such as hepatitis B/C screening or national bowel screening programme?
5. Leadership and ongoing input from experts in Māori and Pacific health, migrant health, gastroenterology, primary health care, public health, microbiology, health service improvement, and epidemiology are vital for the development of a screen‐and‐treat model for Aotearoa.
6. Commitment is needed from funders and the public health system to run a screen‐and‐treat pilot with ongoing feedback informing improvements. This includes developing information technology to generate a system to provide a register, produce invitations, make bookings, carry out recall, and monitor outcomes.

## 11 Conclusions

Aotearoa is well placed to make progress in addressing gastric cancer inequities, and *H. pylori* screen‐and‐treat pilot evaluations are the essential next step. A Māori‐led, equity‐driven approach to implementation will be key for reversing higher rates of gastric cancer in Māori, Pacific, and other high‐risk groups. Investment from funders and the public health system is needed for scaling up and piloting a risk‐based target population *H. pylori* screen‐and‐treat strategy. The findings from a pilot phase will be critical to the success of such a programme.

## Author Contributions

Andrea Teng conceptualized the review, prepared figures and wrote the first draft of this manuscript. James Stanley and Melissa McLeod edited the manuscript, and all authors approved the final draft.

## Funding

The Health Research Council of New Zealand funded time for co‐authors AT and JS working on this manuscript, via the Symbiotic Programme (20/631), HpANZ Study.

## Conflicts of Interest

The authors declare no conflicts of interest.

## Data Availability

Data sharing not applicable to this article as no datasets were generated or analysed during the current study.
